# Guide sheath breakage during endobronchial ultrasonography

**DOI:** 10.1002/rcr2.690

**Published:** 2020-11-20

**Authors:** Takahiro Yanagihara, Masatoshi Yamaoka, Naoki Kawakami, Takuya Onuki, Masaharu Inagaki

**Affiliations:** ^1^ Department of Thoracic Surgery Tsuchiura Kyodo General Hospital Tsuchiura Japan; ^2^ Department of Respiratory Medicine Tsuchiura Kyodo General Hospital Tsuchiura Japan

**Keywords:** EBUS‐GS, endobronchial ultrasonography, guide sheath breakage, instrumental breakage

## Abstract

This report describes a case of guide sheath breakage during endobronchial ultrasonography. While steering the guide sheath in the direction of a peripheral lung nodule using a guiding device/curette, the radiopaque band (RB) attached to the head of the guide sheath dislodged and remained in the peripheral bronchus near the tumour. The band could not be removed endoscopically. As the tumour was diagnosed as a colon cancer metastasis, we performed a partial lung resection to remove the RB and nodule together four months after bronchoscopy.

## Graphical abstract

The radiopaque band attached to the head of the guide sheath dislodged and remained in the peripheral bronchus near the tumour during endobronchial ultrasonography with a guide sheath (EBUS‐GS). The band could not be removed endoscopically. We performed a partial lung resection to remove the radiopaque band and the nodule together four months after EBUS‐GS.

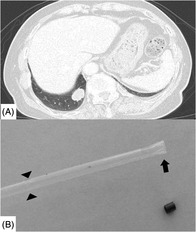

## Introduction

Endobronchial ultrasonography (EBUS) with a guide sheath (EBUS‐GS) is a widely used technique to investigate peripheral pulmonary lesions. Hayama et al. reported that the equipment breakage rate of EBUS‐GS was 0.4%, including only probe breakage but not guide sheath breakage [1]. We present a case of EBUS‐GS breakage in which the radiopaque band (RB) on the head of the guide sheath dislodged inside the body when using a guiding device/curette.

## Case Report

**Figure 1:**
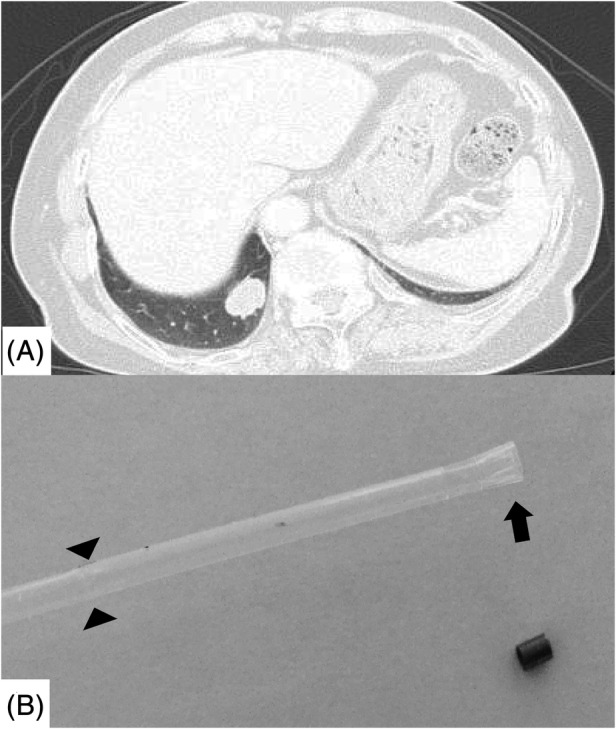
(A) Computed tomography revealing a solid mass of 39 mm in diameter in the right S10 region. (B) The head of the broken guide sheath was expanded (arrow). Traces of buckling were found near the head of the guide sheath (arrow heads).

An 83‐year‐old woman with no medical history was referred to our hospital because of elevated carcinoembryonic antigen. Computed tomography (CT) revealed a solid mass, 39 mm in diameter, in the right lower lobe (RLL) of the lung segment 10 (Fig. 1A). Positron emission tomography showed high ^18^F‐fluorodeoxyglucose uptake in the RLL and descending colon. We performed a diagnostic bronchoscopy (BF‐P290; Olympus Ltd., Japan) with a guide sheath kit (SG‐200C; Olympus Ltd.), an EBUS probe (UM‐S20‐17S; Olympus Ltd.), and a curette (CC‐6DR‐1; Olympus Ltd.). We searched for the bronchi leading to the tumour using a curette and an EBUS probe under fluoroscopic guidance using both the manual mapping method and navigation software (Bf‐NAVI; Olympus Ltd.). We exchanged the EBUS probe for a curette through the guide sheath and manipulated the curette repeatedly to direct the guide sheath to the tumour; the curette was flexed as necessary. It took a long time to choose the correct bronchus because the bronchi were easy to collapse. When we withdrew the guide sheath, the RB detached and lodged in the peripheral bronchus near the tumour, and could not be removed endoscopically. We then found a trace of buckling on the broken guide sheath and expansion of its head (Fig. 1B). After changing to a new device, the patient was diagnosed with the metastasis of colon cancer. The total time was 60 min.

**Figure 2:**
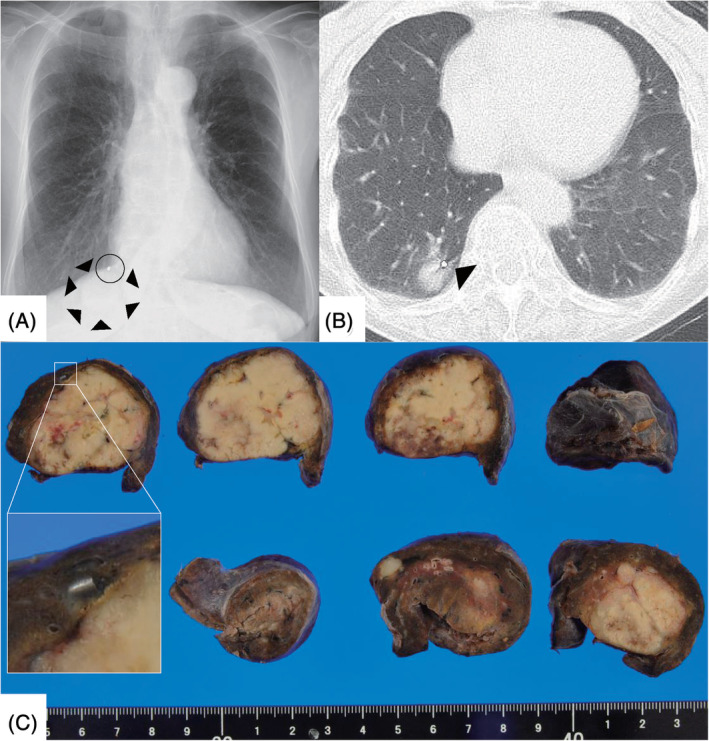
(A) Radiography of the chest showed the radiopaque band(circle) proximal to the original tumour (arrow heads). (B) The radiopaque band was localized to the cranial verge of the original tumour (arrow head). (C) The radiopaque band was identified adjacent to the tumour in the incised specimen.

After a surgical procedure for colon cancer, the patient consulted with our department for surgical resection of the lung metastasis. Chest radiography and CT showed that the RB was localized to the cranial verge of the tumour (Fig. 2A, B). Accordingly, we performed video‐assisted thoracotomy wedge resection of the RLL four months after the bronchoscopy. The post‐operative course was uneventful. The RB was identified adjacent to the tumour in the incised specimen (Fig. 2C).

## Discussion

EBUS‐GS is generally performed to explore peripheral lung nodules using ultrasonography. The guide sheath is made of plastic and is 1.95 mm in diameter. The RB was physically embedded in the inner side of the guide sheath approximately 2.25 mm from its head, and was made of tantalum. In the present case, we attempted to search the peripheral bronchi more selectively using a curette, as described by Kikuchi et al. [2] Consequently, the RB dislodged and remained in the peripheral bronchus. We made inquiries with Olympus Ltd. and considered the following two technical causes: (1) taking the curette in and out from the end of the guide sheath applied a force of flexion to the curette, which may have led to expansion of the head of the guide sheath or (2) the RB may have become lodged in the forceps after passing through the buckled guide sheath. The forceps tend to scratch the internal wall on the peripheral side of the buckled point because of the distortion caused by the buckling; in the present case, we found scratches on the internal wall of the broken guide sheath. These may have facilitated both enlargement of the head of the guide sheath and expulsion of the RB from the guide sheath, causing the RB to fall out. Buckling was expected to occur between the head of the bronchoscope and the entry of the peripheral bronchus where the guide sheath was present, when the bronchoscope was inadvertently withdrawn during the guide sheath insertion into the peripheral bronchus at a steep angle owing to the sudden change in the angle of the head of the bronchoscope. We speculate that manual fixation of the bronchoscope to the patient to prevent its accidental withdrawal during guide sheath insertion could prevent buckling. Moreover, we recommend that the guide sheath be replaced as soon as there is any evidence of instrument stress such as buckling or expansion of the head during examination.

In this case, because of the patient's surgical indications, we managed to remove the RB together with the tumour. Four months elapsed without complications related to the residual RB; we were vigilant for complications such as atelectasis, an impacted mucus plug, and infection, but no such events occurred, given that the small and tube‐like shape of the RB preserved peripheral lung aeration.

In conclusion, bronchoscopists should be aware of the potential small risk of guide sheath breakage and perform bronchoscopy while minimizing the stress of the instruments. In addition, there should be consideration to guide sheath replacement if it is problematic for diagnostic tools to travel through the guide sheath.

### Disclosure Statement

Appropriate written informed consent was obtained for publication of this case report and accompanying images.
